# Therapeutic advances in HR+/HER2- advanced breast cancer after failure of CDK4/6 inhibitor therapy

**DOI:** 10.3389/fonc.2026.1763278

**Published:** 2026-03-13

**Authors:** Mengqi Cui, Junjuan Xiao, Lin Ma, Yue Liang, Jing Liang

**Affiliations:** 1 Department of Oncology, The First Affiliated Hospital of Shandong First Medical University & Shandong Provincial Qianfoshan Hospital, Shandong Key Laboratory of Rheumatic Disease and Translational Medicine, Shandong Lung Cancer Institute, Jinan, China; 2 Breast Center, The Second Qilu Hospital of Shandong University, Jinan, China

**Keywords:** advanced breast cancer, CDK4/6i treatment failure, ER-positive disease, follow-up treatment, novel targeted therapies

## Abstract

Breast cancer represents the most frequently diagnosed malignancy in women, with the hormone receptor-positive/HER2-negative (HR+/HER2-) subtype being the most prevalent. For advanced HR+/HER2- breast cancer, the combination of cyclin-dependent kinase 4/6 inhibitors (CDK4/6i) with endocrine therapy has become the established first-line standard, significantly prolonging both median progression-free survival (mPFS) and overall survival (OS). Nevertheless, the majority of patients eventually develop resistance, leading to disease progression. The underlying mechanisms of resistance are multifactorial, involving dysregulated cell cycle control, reprogramming of signaling pathways, and remodeling of the tumor microenvironment (TME). At present, there is no standardized treatment strategy for overcoming CDK4/6i resistance. This article systematically reviews current post-CDK4/6i therapeutic strategies, including next-generation endocrine therapies (e.g., oral SERDs), targeted agents directed at the PI3K/AKT/mTOR axis, AKT inhibitors, and antibody–drug conjugates (ADCs), and discusses potential future therapeutic directions.

## 1 Introduction

### 1.1 Epidemiological and clinical characteristics of HR+/HER2- breast cancer

Based on the latest international cancer statistics, breast cancer continues to rank as the most frequently diagnosed malignancy in women, imposing a significant mortality burden worldwide (1). Surgical intervention for advanced breast cancer (ABC) is generally ineffective, and these patients typically have a poor prognosis (2). Among breast cancer subtypes, the HR+/HER2- subtype accounts for 60-70% of cases, is more common in postmenopausal women, and its incidence gradually increases with age (median age at diagnosis is 62 years). The disease progression is relatively slow but exhibits significant heterogeneity: Luminal A tumors are generally associated with a more favorable long-term outcome and lower recurrence risk, whereas Luminal B tumors display higher proliferative activity and a greater likelihood of earlier relapse. This biological and clinical distinction has been consistently demonstrated in molecular classification studies (3). HR+/HER2- subtype primarily metastasizes to the bones (70%), followed by lymph nodes, lungs, and liver. Among these, visceral metastasis (especially liver metastasis) indicates a poor prognosis (4). The current standard first-line treatment for metastatic HR+ breast cancer involves CDK4/6i combined with endocrine therapy (2, 5–8). Although this regimen can extend median PFS to 24–28 months, acquired drug resistance is inevitable (9). After resistance develops, disease progression accelerates, and subsequent treatment options become limited (3, 10). Therefore, selecting treatments after disease progression poses notable clinical challenges.

### 1.2 The clinical status of CDK4/6i

CDK4/6i represent the gold standard for first-line treatment of HR+/HER2- advanced breast cancer. They exert anti-tumor effects by precisely targeting the cell cycle regulatory machinery. The core mechanism involves selective inhibition of cyclin-dependent kinases 4 and 6, which blocks retinoblastoma protein (RB) phosphorylation, thereby inhibiting the release of the transcription factor E2F. This ultimately causes tumor cell cycle arrest at the G1 phase and effectively prevents entry into the S phase of DNA synthesis. CDK4/6i exhibit significant synergy with endocrine therapy, can reverse endocrine resistance, and block alternative proliferative signaling pathways (11). In clinical practice, this drug class has demonstrated significant efficacy (pivotal Phase III studies (12–14) have shown that the combination regimen nearly doubles PFS and improves response rates compared to endocrine therapy alone), provides comprehensive survival benefits (prolonged OS and consistent efficacy across all subgroups), and has a favorable safety profile [the main adverse event is reversible neutropenia, with mild non-hematologic toxicity and low treatment discontinuation rates (15–17)]. Moreover, they significantly delays the need for chemotherapy.

Compared with traditional chemotherapy, CDK4/6i therapy is more individualized (based on HR/HER2 status) and offers advantages such as specific tumor targeting, mild and reversible myelosuppression, and absence of alopecia (15). Relative to other targeted therapies, it is applicable to a broader patient population (without requiring biomarker screening) and has fewer drug-drug interactions, making it the preferred option in clinical practice.

## 2 The resistance mechanism of CDK4/6i

Although CDK4/6i combined with endocrine therapy have become the standard first-line treatment for advanced estrogen receptor–positive (ER+) breast cancer, the development of drug resistance ultimately limits long-term therapeutic benefit. The molecular basis of both primary (18) and acquired resistance is complex and multifactorial, involving alterations in core cell-cycle regulators, activation of compensatory signaling pathways, and dynamic interactions with the tumor microenvironment. Mechanistically, these resistance processes can be broadly categorized into three principal classes: (1) dysregulation of cell-cycle control, (2) activation of bypass growth and survival pathways, and (3) tumor microenvironment–mediated and phenotypic adaptive changes. The following subsections are organized according to this framework to clarify cause–effect relationships and highlight their therapeutic implications. Understanding this structured hierarchy is essential for linking resistance biology to rational therapeutic sequencing strategies.

### 2.1 Rb and Rb/E2F signal conduction

When patients undergo endocrine therapy (e.g., fulvestrant) or targeted therapy (e.g., CDK4/6i), the vast majority of cancer cells dependent on original survival signaling pathways (such as estrogen receptor signaling and cell cycle pathways) are effectively suppressed or eliminated. Under such therapeutic pressure, the APOBEC3 system becomes aberrantly activated, enabling cancer cells to persist. This system significantly enhances tumor genetic diversity by introducing widespread random mutations throughout the genome. Consequently, drug treatment exerts selective pressure, leading to the enrichment of drug-resistant clones (19) that harbor specific mutations in key genes (e.g., RB1 and ESR1) and thereby acquire a survival advantage, ultimately resulting in treatment failure. This phenomenon reflects treatment-imposed selective pressure that promotes the expansion of resistant tumor subclones.

RB dysfunction in cancer cells, therefore, fundamentally bypasses the CDK4/6-RB axis, which is the primary pathway for cell cycle control. Such cells are intrinsically insensitive to CDK4/6i, representing a primary mechanism of drug resistance (20). Among various resistance mechanisms, the functional status of the RB1 gene plays a central role (21). The RB protein acts as a critical “brake” protein downstream of CDK4/6 kinase, controlling the cell cycle transition from the G1 phase to the S phase.

When the RB1 gene is inactivated due to mutation, cancer cells can bypass CDK4/6i-induced cell cycle blockade and resume proliferation. Moreover, RB1-inactivating mutations can exist in a “polyclonal” manner within a patient, meaning that multiple cellular subpopulations carry independent RB1 mutations (22). This polyclonal resistance confers greater stability to the drug-resistant phenotype, making it more difficult to eradicate with subsequent therapies. Studies have confirmed that intact RB1 function is a necessary condition for CDK4/6i efficacy, while its loss represents a sufficient condition for resistance.

Beyond genetic mutations, epigenetic mechanisms also play a critical role—notably, HDAC5 function (23). Even when CDK4/6i successfully activate the RB protein, fully functional RB-mediated growth suppression still depends on HDAC5. Thus, although HDAC5 loss does not degrade the RB protein itself, it leads to incomplete RB function. Consequently, CDK4/6i resistance can still arise in tumors with intact RB protein.

Another key biomarker associated with resistance is overexpression of p16 protein. When cancer cells exhibit abnormally high p16 levels (24), this usually does not reflect active inhibition of proliferation, but rather a compensatory feedback response. It often indicates abnormally strong upstream signaling in the RB pathway (e.g., Cyclin D1 amplification or overactivation), which enables cells to proliferate despite high levels of endogenous inhibitory signals, thereby diminishing the effect of exogenous CDK4/6 inhibitors.

Together, these mechanisms provide a solid theoretical foundation and practical direction for advancing individualized therapy: they may help avoid ineffective treatments in patients likely to exhibit resistance, and support the development of novel combination strategies to overcome such resistance.

### 2.2 Cyclin E and CDK2

In a normal cell cycle, the transition of cells from the G1 phase to the S phase depends on a cascade reaction. The growth signal first activates the Cyclin D-CDK4/6 complex, which acts as a “start switch” to partially phosphorylate the Rb protein. The released E2F transcription factor subsequently initiates the expression of S-phase related genes (including Cyclin E (25)). The newly synthesized Cyclin E then combines with CDK2 to form a complex, leading to hyperphosphorylation of Rb, which completely releases the brake and allows the cells to enter the S phase.

The design of CDK4/6i precisely interrupts this process by inhibiting the initial “start switch” Cyclin D-CDK4/6 complex, thereby arresting cells in the G1 phase (26). However, when tumor cells exhibit abnormally high expression of Cyclin E, drug resistance can occur. This overexpression may result from amplification of the CCNE1 gene or abnormal activation of other signaling pathways. As a result, abundant Cyclin E accumulates and binds to CDK2, forming active Cyclin E-CDK2 complexes (27). These complexes no longer depend on upstream CDK4/6 signaling and can directly and effectively phosphorylate Rb protein, driving the cell cycle into S phase. Crucially, this process establishes a dangerous reinforced positive feedback loop: the released E2F further promotes Cyclin E synthesis, leading to more Cyclin E-CDK2 complexes, which in turn phosphorylate Rb and release E2F more completely. Once this cycle is established, the cell progression becomes entirely independent of CDK4/6. Moreover, the highly active Cyclin E-CDK2 complex significantly accelerates G1 progression, shortens the “sensitive window” for drug action, and enables cancer cells to rapidly bypass the cell cycle checkpoint. In summary, through Cyclin E overexpression and subsequent CDK2 activation, tumor cells evade CDK4/6 inhibition and sustain proliferation. Notably, CDK2 activation represents a convergent downstream node across multiple resistance pathways. Several upstream alterations discussed below—such as FAT1 loss, TROJAN overexpression, PI3K pathway activation, and FGFR signaling—ultimately enhance Cyclin E–CDK2 activity, reinforcing Rb phosphorylation and enabling CDK4/6-independent cell cycle progression. To avoid redundancy, subsequent sections focus on pathway-specific mechanisms that feed into this shared CDK2-driven bypass. While direct cell-cycle alterations constitute one major class of resistance, additional pathways contribute indirectly by activating alternative proliferative signaling cascades, as discussed below.

### 2.3 TP53

CDK4/6i causes sensitive breast cancer cells to undergo cell cycle arrest and enter a dormant state, but the ultimate outcome of this state mainly depends on the functional integrity of the TP53 gene. In cancer cells with normal TP53 function, the drugs activate the p53 protein (28), initiating the irreversible process of cellular senescence, which leads to permanent loss of proliferative capacity, thereby achieving a lasting therapeutic effect. In contrast, in cancer cells lacking functional TP53, drug-induced dormancy is reversible and represents a “standby” state. During dormancy, such cells activate alternative survival pathways such as mTOR, accumulate potential for drug resistance, and once conditions permit, can re-enter the proliferation cycle, leading to disease recurrence. This mechanism indicates that the absence of TP53 does not directly cause drug resistance but enables cancer cells to leverage drug-induced dormancy as a springboard for recurrence. Therefore, for patients with TP53 mutations, combining CDK4/6i with mTOR inhibitors (29) is expected to effectively prevent tumor recurrence by blocking survival pathways during the dormant period.

### 2.4 FAT1 deficiency

A study analyzed the clinical data of 348 ER+ breast cancer patients who had received CDK4/6i treatment and found that patients carrying FAT1 or RB1 loss-of-function mutations had significantly shortened progression-free survival, suggesting that the failure of these two genes is closely related to drug resistance. In order to confirm that FAT1 deficiency is the cause of drug resistance, the researchers in the lab, knocked out the FAT1 gene in CDK4/6i-sensitive breast cancer cells, successfully induced resistant phenotypes, and CDK6 protein in cells was observed at the same time higher level of specificity. Mechanism to explore further revealed that FAT1 is the Hippo signaling pathway of a positive regulatory factor. When FAT1 is absent, the Hippo pathway is inhibited, leading to the activation of its downstream oncoproteins YAP/TAZ (30) and their entry into the cell nucleus. These proteins then bind to the TEAD transcription factor, directly initiating the transcription of the CDK6 gene. The team found that in FAT1-deficient drug-resistant cells, targeting the downstream of the Hippo pathway by inhibiting the expression or function of YAP or TAZ could effectively reduce CDK6 levels and make the cells sensitive to CDK4/6i again, which provides important experimental evidence for future combination treatment regimens.

### 2.5 TROJAN high expression

The abnormally high expression of long non-coding RNA TROJAN (no official expansion, retained as the original gene symbol) (31) in ER+ breast cancer is a key initiating factor leading to resistance to CDK4/6i. Its mechanism of action involves directly targeting and disrupting a key transcriptional repression complex: TROJAN binds to the ZMYND8 protein, acting as a “molecular wedge” that disrupts the stable dimer formed with ZNF592, thereby releasing the transcriptional inhibition of the CDK2 gene by this complex. As the “brake” on the CDK2 gene is removed, the mRNA and protein expression levels rise significantly and continuously. This enables cancer cells to activate the abundant CDK2 already present within the cells, even when the main cell cycle driver CDK4/6 is effectively blocked by CDK4/6i treatment. Elevated CDK2 activity promotes CDK4/6-independent cell cycle progression, consistent with the convergent bypass mechanism described above. This discovery not only reveals a novel mechanism of drug resistance but also directly suggests that combining CDK2 inhibitors or directly targeting TROJAN itself represents a potential future strategy to overcome such resistance. While Sections 2.1–2.5 illustrate resistance mechanisms driven primarily by direct dysregulation of core cell-cycle regulators, the following sections focus on alternative growth and survival signaling pathways that indirectly converge on CDK2 activation and promote CDK4/6-independent proliferation.

### 2.6 PI3K/AKT/mTOR signaling pathway

In hormone receptor-positive breast cancer, aberrant activation of the PI3K/AKT/mTOR signaling pathway (32) represents a core mechanism underlying treatment resistance. Under physiological conditions, this pathway is tightly regulated by factors such as PTEN; in tumors, however, it is frequently constitutively activated due to PIK3CA mutations or PTEN deficiency, enabling cancer cells to receive proliferative signals through intrinsic routes and thereby develop drug resistance even after exogenous endocrine therapy blocks estrogen signaling (33, 34). PTEN loss holds particular clinical significance, as it not only leads to endocrine therapy resistance but can also cause dual resistance to both CDK4/6i and PI3K inhibitors. Mechanistically, persistent PI3K/AKT/mTOR activation enhances cyclin D1 expression and reinforces CDK2-driven proliferation, thereby converging on the shared bypass mechanism described in Section 2.2. Moreover, the signal redundancy resulting from PTEN deficiency impairs the ability of a single PI3K inhibitor to effectively block overall pathway activity. Consequently, PTEN loss establishes a highly active and redundant PI3K signaling environment (35), contributing to cross-resistance and explaining why patients with PTEN deficiency respond poorly to PI3K inhibitors after CDK4/6i treatment.

### 2.7 Other signaling pathways

**Figure 1:**
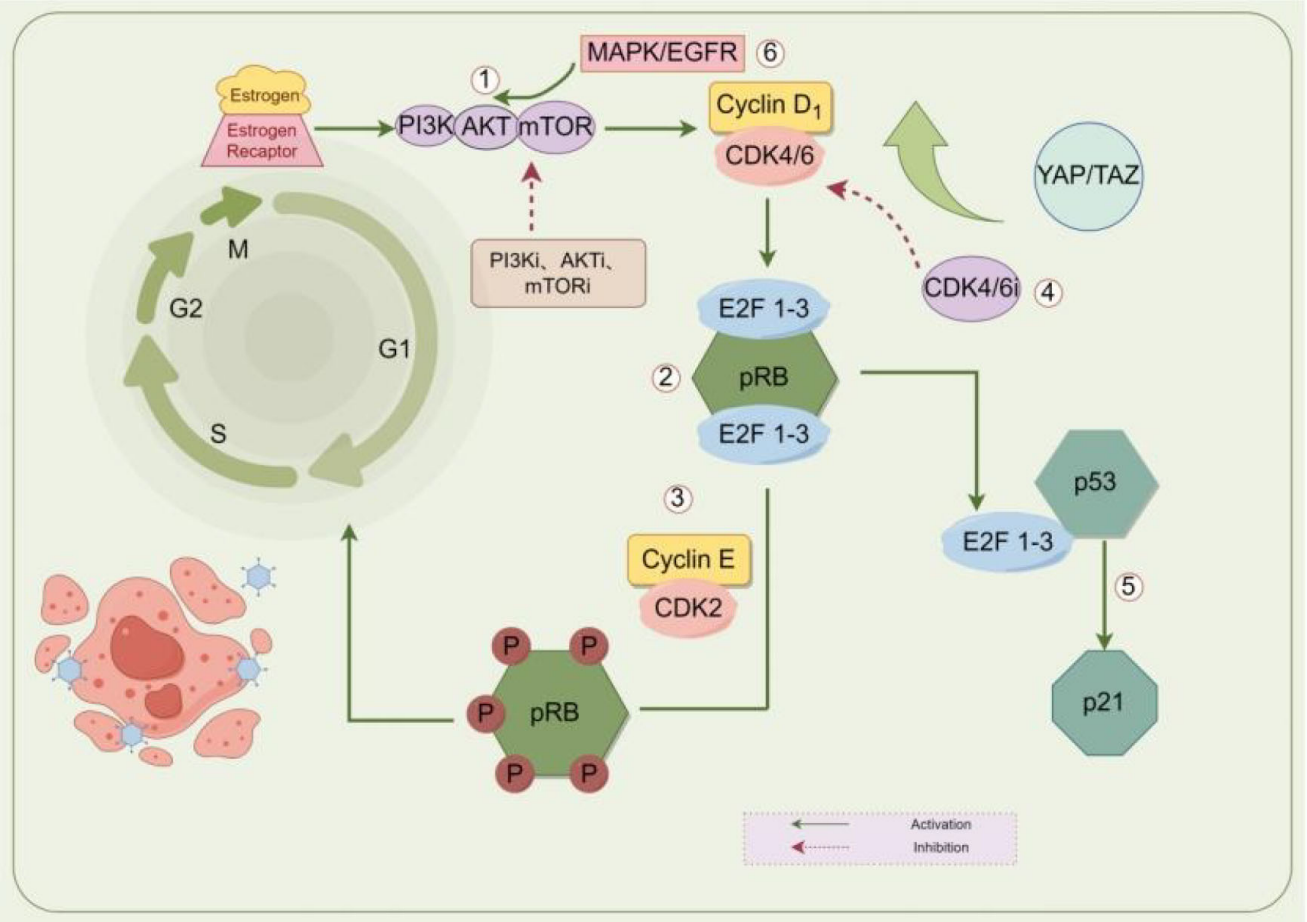
Mechanisms underlying CDK4/6i resistance. Main regulatory mechanism: ①Abnormal activation of the PI3K/AKT/mTOR pathway and upregulation of Cyclin D1 expression; ②When a deletion or inactivation mutation occurs in the RB1 gene of the RB protein, CDK4/6i cannot prevent Rb phosphorylation; ③The Cyclin E-CDK2 complex is abnormally activated, drive the cell cycle process, thereby bypassing the dependence on CDK4/6; ④Amplification or overexpression of CDK4/6, thereby drowning out CDK4/6i; ⑤The destruction of the p53 signaling pathway makes it impossible to effectively induce aging, thereby leading to drug resistance; ⑥Mutations or activation of RAS family genes. CDK, cyclin-dependent kinase; RB, retinoblastoma protein; mTOR, mammalian target of rapamycin; AKT, Ak strain Transforming; PI3K, phosphoinositide-3-kinase;ERK,extracellular signal regulated kinases; YAP, Yes associated protein; TAZ, transcriptional coactivator with PDZ binding motif; E2F, E2Factor; EGFR, Epidermal Growth Factor Receptor; MAPK, Mitogen Activated Protein Kinase. **Figure 1** was created using FigDraw.

Cell clones with aberrant FGFR signaling activity acquire selective survival advantages. The core mechanism of drug resistance lies in the ability of activated FGFR signaling to stimulate two key downstream pathways, MAPK/ERK and PI3K/AKT, which collectively upregulate the cell cycle protein Cyclin E. The resulting increase in CDK2 activity reinforces the CDK4/6-independent proliferative program described above. Additionally, FGFR pathway activation (36) can induce epithelial-mesenchymal transition (EMT), shifting tumor cells toward a more primitive and aggressive state, thereby reducing dependency on CDK4/6-mediated control and promoting alternative proliferative circuitry. These mechanisms reinforce each other through complex positive feedback loops, forming a flexible signaling network that enables tumor cells to sustain proliferation via FGFR signaling hubs even under CDK4/6 inhibition (**Figure 1**). Collectively, these resistance routes can be organized according to their biological drivers and corresponding therapeutic vulnerabilities. To provide a concise overview linking resistance mechanisms to potential treatment strategies, **Table 1** summarizes the major resistance categories and their clinical implications.

**Table 1:** Major mechanisms of resistance to CDK4/6 inhibitors and their therapeutic implications.

Resistance category	Key molecular alteration	Biological consequence	Potential therapeutic strategy
Cell cycle escape	RB loss, Cyclin E amplification	CDK4/6 bypass	Chemotherapy, alternative kinase targets
Estrogen pathway reactivation	ESR1 mutation	Ligand-independent ER activation	Oral SERDs
PI3K/AKT/mTOR activation	PIK3CA mutation, PTEN loss	Survival pathway upregulation	PI3K/AKT inhibitors
HER2/MAPK bypass	HER2 amplification	Parallel growth signaling	HER2-targeted therapy
TME remodeling	Immune suppression	Reduced immune surveillance	Immunotherapy combinations

RB, retinoblastoma protein; ER, estrogen receptor; SERD, selective estrogen receptor degrader; PI3K, phosphoinositide-3-kinase; AKT, Ak strain Transforming kinase; mTOR, mammalian target of rapamycin; PIK3CA, phosphatidylinositol-4,5-bisphosphate 3-kinase catalytic subunit alpha; PTEN, phosphatase and tensin homolog; MAPK, mitogen-activated protein kinase; TME, tumor microenvironment.

These resistance mechanisms provide a biological rationale for the selection of subsequent therapeutic strategies. Activation of the PI3K/AKT/mTOR signaling axis, frequently driven by PIK3CA mutations or PTEN loss, supports the clinical use of PI3K, AKT, or mTOR inhibitors following CDK4/6i progression. Similarly, the emergence of ESR1 mutations, particularly after aromatase inhibitor exposure, underlies the development of next-generation selective estrogen receptor degraders (SERDs) designed to overcome ligand-independent ER activation. Alterations that restore cell-cycle progression through CDK2 signaling, Cyclin E amplification, or RB pathway disruption suggest a reduced likelihood of benefit from continued CDK4/6 blockade, thereby favoring strategies targeting parallel pathways or utilizing non-endocrine mechanisms. In addition, tumor evolution toward HER2-low phenotypes or increased genomic instability may partly explain the efficacy of antibody–drug conjugates (ADCs) in heavily pretreated populations. Therefore, understanding the molecular context of resistance is essential for rational therapeutic sequencing after CDK4/6 inhibitor failure.

## 3 Existing treatment strategies after the failure of CDK4/6i

### 3.1 Endocrine therapy combined with targeted drugs

ET aims to block the proliferation of breast cancer cells driven by estrogen. For postmenopausal HR+ breast cancer, the main therapies include: selective estrogen receptor modulators (SERMs, such as tamoxifen), aromatase inhibitors (AIs, such as letrozole, etc.) to reduce estrogen production, and selective estrogen receptor degraders (SERDs, such as fulvestrant). When HR+/HER2- ABC patients develop resistance to CDK4/6i, ET combined with targeted drugs becomes a key strategy. Research has confirmed that for specific molecular abnormalities (such as PIK3CA mutations, ESR1 mutations, or alterations in the PIK3CA/AKT/PTEN pathway), the combination of PI3K inhibitors (such as alpelisib), novel oral SERDs (such as elacestrant), or AKT inhibitors (such as capivasertib) can significantly improve PFS. For patients with multi-drug resistance, everolimus combined with endocrine therapy still holds value.

#### 3.1.1 Fulvestrant + mTOR inhibitor

In patients with HR+/HER2- advanced breast cancer who have progressed on CDK4/6i therapy, the combination of fulvestrant and everolimus demonstrates a synergistic antitumor effect through dual blockade of the ER signaling pathway (37) and the PI3K/AKT/mTOR pathway. Fulvestrant potently downregulates and degrades ERα, thereby inhibiting estrogen-dependent proliferation, while everolimus counteracts pathway-mediated resistance via mTORC1 inhibition. The BOLERO-2 study confirmed that the combination therapy significantly prolongs median progression-free survival compared with single-agent therapy (7.8 vs. 3.2 months), increases the objective response rate by nearly sevenfold, and shows consistent benefits across all subgroups (38, 39). Another phase II study also demonstrated a doubling of median progression-free survival in the combination group (10.3 vs. 5.1 months) and a significantly improved clinical benefit rate (**Table 2**).

**Table 2:** Key clinical trials in HR+/HER2− advanced breast cancer, grouped by line of therapy.

Category	Study (NCT)	Phase	Treatment	N	mPFS (months)	HR (95% CI)	OS (months)
A. First-line CDK4/6 inhibitor registration trials							
CDK4/6i	PALOMA-2 (NCT01740427)	Phase III	Palbociclib + Letrozole vs Placebo + Letrozole	666	27.6 vs 14.5	0.56 (95% CI: 0.46–0.69; P < 0.001)	53.9 vs 51.2
CDK4/6i	MONALEESA-2 (NCT01958021)	Phase III	Ribociclib + Letrozole vs Placebo + Letrozole	668	25.3 vs 16.0	0.57 (95% CI: 0.46–0.70; P < 0.001)	63.9 vs 51.4
CDK4/6i	MONARCH-3 (NCT02246621)	Phase III	Abemaciclib + NSAI vs Placebo + NSAI	726	20.5 vs 12.8	0.59 (95% CI: 0.48–0.73; P < 0.001)	53.7 vs 41.5
B. Trials conducted after CDK4/6i progression							
AKT inhibitor	CAPItello-291 (NCT04305496)	Phase III	Capivasertib + Fulvestrant vs Placebo + Fulvestrant	708	7.3 vs 3.6	0.60 (95% CI: 0.51–0.71; P < 0.001)	-
mTOR inhibitor	BOLERO-2 (NCT00863655)	Phase III	Everolimus + Exemestane vs Placebo + Exemestane	724	6.9 vs 2.8	0.45 (95% CI: 0.38–0.54; P < 0.001)	12.6 vs 10.4
PI3K inhibitor	BYLieve (NCT03056755)	Phase II	Alpelisib + Fulvestrant	127	8	-	-
PI3K inhibitor	SOLAR-1 (NCT02437318)	Phase III	Alpelisib + Fulvestrant vs Placebo + Fulvestrant	572	11.1 vs 5.7	0.65 (95% CI: 0.50–0.85; P < 0.001)	39.3 vs 31.4
Novel SERD	EMERALD (NCT03778931)	Phase III	Elacestrant vs Standard Endocrine Therapy	478	3.8 vs 1.9	0.55 (95% CI: 0.39–0.77; P < 0.001)	-
Novel SERD	SERENA-2 (NCT04214288)	Phase II	Camizestrant vs Fulvestrant	240	7.2/7.7 vs 3.7	0.65 (95% CI: 0.50–0.85)	-
Novel SERD	AMEERA-3 (NCT04059484)	Phase II	Amcenestrant vs Physician’s Choice ET	290	3.6 vs 3.7	1.05 (95% CI: 0.79–1.40; P = 0.64)	-
Trop-2 ADC	TROPiCS-02 (NCT03901339)	Phase III	Sacituzumab Govitecan vs Physician’s Choice Chemotherapy	543	5.5 vs 4.0	0.66 (95% CI: 0.53–0.83; P < 0.001)	14.4 vs 11.2
HER2 ADC	DESTINY-Breast04 (NCT03734029)	Phase III	Trastuzumab Deruxtecan vs Physician’s Choice Chemotherapy	557	10.1 vs 5.4	0.50 (95% CI: 0.40–0.63; P < 0.001)	23.9 vs 17.9

mPFS, median progression-free survival; OS, overall survival; CI, confidence interval; NSAI, non-steroidal aromatase inhibitor; ET, endocrine therapy; ADC, antibody–drug conjugate.

However, this regimen is associated with a more prominent adverse event profile (38), including stomatitis (60% all-grade, 8% grade 3–4), pneumonitis (3.6% grade 3–4), metabolic abnormalities, and an increased risk of infection, leading some patients to require dose adjustments or early treatment discontinuation. In clinical practice, enhanced monitoring and management are essential to optimize safety. Following CDK4/6 inhibitor treatment, the combination of fulvestrant and everolimus remains an important therapeutic option, particularly in patients with abnormalities in the PI3K/AKT/mTOR pathway, though its relatively narrow therapeutic window poses a challenge among newer targeted agents.

#### 3.1.2 Fulvestrant + PI3K inhibitor

In patients with HR+/HER2- advanced breast cancer who failed CDK4/6 inhibitor treatment, fulvestrant combined with the PI3K inhibitor alpelisib demonstrated significant anti-tumor activity by dual blocking of the ER and PI3K/AKT/mTOR pathways. Alpelisib can specifically inhibit the excessive activation (38–40) of the pathway caused by PIK3CA mutations, thereby reversing endocrine drug resistance.

Key phase III study confirms that SOLAR -1 (40) in PIK3CA mutations in patients with combined treatment single-agent significantly prolong the median progression-free survival (11.0 vs. 5.7 months), the objective response rate 3 times, and sustained efficacy was observed in patients previously treated with CDK4/6i. The BYLieve trial further verified its efficacy, and all subgroups, including patients with visceral metastases, benefited consistently. However, the adverse reactions of alpelisib were significant. 64% of patients experienced hyperglycemia (36% of which were grade 3 or above), and other common reactions included diarrhea, rash and nausea. Clinically, it is necessary to focus on monitoring the blood glucose of high-risk patients and intervene in a timely manner. Compared with mTOR inhibitors, alpelisib has better predictive efficacy for patients with PIK3CA mutations, but its toxicity management is more complex. This protocol offers an effective option for patients (40, 41) resistant to CDK4/6i and highlights the necessity of PIK3CA mutation detection (42) in precision treatment.

#### 3.1.3 New selective estrogen receptor degraders

ESR1 mutations represent a common mechanism of endocrine therapy resistance in metastatic breast cancer, occurring in approximately 36% of cases (43, 44). Oral SERDs have emerged as a key strategy to overcome this form of resistance by binding to and degrading the estrogen receptor. Currently, several oral SERDs under clinical development include elacestrant, camizestrant, amcenestrant, and giredestrant, among others.

Among these, elacestrant not only fully degrades the ERα protein but also inhibits ESR1 activation and ERα expression (44), demonstrating efficacy even in CDK4/6i-resistant cells. Its unique molecular structure contributes to a more comprehensive blockade of the ER signaling pathway. In the Phase III EMERALD study, which enrolled 478 patients with advanced breast cancer (45) previously treated with CDK4/6i and at least one line of endocrine therapy, results showed that the elacestrant group achieved significantly higher progression-free survival rates at both 6 and 12 months compared to standard endocrine therapy, both in the overall population and in the ESR1-mutant subgroup (46, 47).

Regarding safety, common adverse events associated with oral SERDs include menopausal symptoms such as hot flashes, night sweats, and arthralgia, which may impact patients’ quality of life. In summary, oral SERDs (48) effectively suppress tumor growth by degrading the estrogen receptor, offering a novel therapeutic option for patients with advanced breast cancer harboring ESR1 mutations following prior CDK4/6i-based treatment.

### 3.2 Chemotherapy and antibody-drug conjugates

#### 3.2.1 Single-agent chemotherapy

In patients with HR+/HER2-ABC who have progressed on CDK4/6i therapy, monotherapy such as capecitabine or eribulin remains an important treatment option, particularly for those with rapid disease progression or significant symptoms. Capecitabine, an oral fluoropyrimidine precursor, is converted to 5-fluorouracil (5-FU) within tumor cells by thymidine phosphorylase, thereby interfering with DNA synthesis and RNA function. Its mechanism of action is distinct from that of endocrine and targeted therapies, which may allow it to remain effective in patients with CDK4/6i resistance. Eribulin, a microtubule dynamics inhibitor, exerts antitumor effects by suppressing microtubule growth and disrupting mitotic spindle formation. Its unique mechanism also demonstrates efficacy in taxane-resistant tumors.

Currently, single-agent chemotherapy maintains a fundamental role after CDK4/6i failure, though the median progression-free survival generally ranges from 4 to 6 months. The advantages of these chemotherapeutic agents include a relatively rapid onset of action, independence from specific molecular markers, and extensive clinical experience. However, they also present notable limitations. Capecitabine is frequently associated with hand-foot syndrome (45–50%) and diarrhea (30%), while eribulin commonly leads to neutropenia (50–60%) and peripheral neuropathy (20–30%). Compared with targeted therapies, chemotherapy tends to have a more pronounced negative impact on quality of life, and validated predictive biomarkers for efficacy are currently lacking.

The current NCCN guidelines (2024) continue to include single-agent chemotherapy as a recommended option after CDK4/6i failure, especially for patients experiencing visceral crisis or those intolerant to targeted agents. Nevertheless, with the emergence of novel therapeutics, the role of chemotherapy in the treatment landscape is being re-evaluated. When making clinical decisions, it is essential to integrate multiple factors—including disease burden, prior treatment toxicities, molecular profile, and patient preferences—to balance therapeutic efficacy with quality of life.

#### 3.2.2 TROP-2 ADC

For patients with HR+/HER2- advanced breast cancer who have progressed after CDK4/6i therapy, the TROP-2–targeting antibody–drug conjugate (ADC) sacituzumab govitecan (SG) has demonstrated notable efficacy in later-line treatment. SG consists of a humanized anti–TROP-2 antibody conjugated to the topoisomerase I inhibitor SN-38 via a hydrolyzable linker. Upon binding to TROP-2–expressing tumor cells, the ADC is internalized, and SN-38 is released intracellularly, resulting in DNA damage and apoptosis; it also exerts a potent bystander killing effect on neighboring tumor cells.

The phase III TROPiCS-02 study enrolled 543 heavily pretreated patients with advanced disease (49–52). Results showed that, compared with physician’s choice of chemotherapy, SG significantly improved median progression-free survival (5.5 vs. 4.0 months) and overall survival (14.4 vs. 11.2 months), while nearly doubling the objective response rate. Importantly, treatment benefit was observed regardless of TROP-2 expression level.

The most common adverse events associated with SG include myelosuppression (neutropenia occurring in 72% of patients, with 51% being grade 3–4), diarrhea (64%), and other gastrointestinal toxicities such as nausea and vomiting. There is also a risk of interstitial lung disease (approximately 2%), warranting close clinical monitoring. With appropriate supportive care and management, the safety profile of SG is generally considered manageable.

#### 3.2.3 HER2-low ADC

Trastuzumab Deruxtecan (T-DXd), as a representative ADC for HER2-low breast cancer, enables precise treatment through a unique mechanism. This agent is composed of trastuzumab covalently linked to the topoisomerase I inhibitor deruxtecan via a cleavable linker. After binding to the HER2 receptor, the ADC is internalized into cells, where cytotoxic payloads are released, resulting in DNA damage and apoptosis (53).

The DESTINY-Breast04 study established the efficacy of T-DXd in HER2-low advanced breast cancer. This trial enrolled 557 patients who had previously received one or two lines of chemotherapy. Results demonstrated that T-DXd significantly prolonged median progression-free survival (10.1 months vs. 5.4 months) and overall survival (23.9 months vs. 17.5 months) compared with physician’s choice of chemotherapy. The objective response rate was more than three times higher (52.6% vs. 16.3%).

Regarding safety, grade 3–4 treatment-related adverse events occurred in 56% of T-DXd–treated patients. The most notable risk was interstitial lung disease (16.5% all-grade; 3.5% grade 3–5), which necessitates careful monitoring and early intervention.

Compared with TROP-2–directed ADCs, T-DXd demonstrates superior efficacy in the HR+ population but carries a higher risk of ILD. In contrast to PI3K/mTOR inhibitors, T-DXd acts independently of specific gene mutations but involves higher treatment costs. With the ongoing development of ADC therapies, their role in advanced breast cancer continues to expand, offering patients new therapeutic options (54). At the same time, their use demands more sophisticated toxicity management and rational sequencing strategies.

### 3.3 Emerging targeted therapy

#### 3.3.1 AKT inhibitor

The PI3K/AKT signaling pathway is one of the most frequently dysregulated pathways in human cancers, with alterations observed in approximately 38% of cancer patients. In breast cancer, over 50% of patients harbor related genetic variations, such as PIK3CA mutations or PTEN deletions. Aberrant activation of this pathway is closely associated with tumor development and resistance to chemotherapy (55).Among targeted agents for this pathway, the oral AKT inhibitor capivasertib acts by inhibiting the phosphorylation of downstream AKT substrates. Both the Phase II FAKTION study and the pivotal Phase III CAPItello-291 trial (56) confirmed that in patients previously treated with CDK4/6i, the combination of capivasertib and fulvestrant significantly improved progression-free survival and nearly tripled the objective response rate. Based on these results, capivasertib received FDA approval in 2023 and is recommended in the 2024 NCCN guidelines. Its intermittent dosing schedule—four days on and three days off—contributes to improved treatment tolerance. Common adverse events include diarrhea, rash, and hyperglycemia, most of which are manageable (57, 58).

Compared with the PI3K inhibitor alpelisib, capivasertib is associated with a lower risk of hyperglycemia and is not restricted to patients with PIK3CA mutations. Relative to mTOR inhibitors, it offers more precise targeting and a lower incidence of stomatitis.

#### 3.3.2 CDK2 inhibitors (targeting high expression of Cyclin E)

In patients with HR+/HER2- advanced breast cancer who have failed CDK4/6i treatment, CDK2 inhibitors targeting the high-expression subpopulation of Cyclin E represent a potential precision treatment strategy (59).Its mechanism of action stems from an in-depth understanding of the resistance mechanism of CDK4/6 inhibitors: When tumor cells are continuously inhibited by CDK4/6, CDK2 is activated by up-regulating the expression of Cyclin E (60), thereby bypassing the CDK4/6-RB-dependent cell cycle regulation and driving tumor cell proliferation. CDK2 inhibitors re-establish cell cycle arrest by selectively blocking the kinase activity of the Cyclin E-CDK2 complex, inhibiting abnormal phosphorylation of RB protein and release of E2F transcription factor (61). This mechanism has shown significant anti-tumor activity against CDK4/6 inhibitor resistance models in preclinical studies. PF-07104091, currently in the clinical development stage, as a highly selective CDK2 inhibitor, demonstrated an objective response rate (ORR 24%) for HR+ breast cancer patients with high Cyclin E expression in the early data of the Phase I clinical trial (NCT04553133). Moreover, the median progression-free survival reached 5.8 months, and this efficacy was particularly prominent in patients who had previously received CDK4/6 inhibitor treatment. From a clinical perspective, the value of CDK2 inhibitors lies (62) in their targeting of clear drug resistance biomarkers (overexpression of Cyclin E), providing precise treatment options for the Cyclin E high-expression subpopulation, which accounts for approximately 30-40% of CDK4/6 inhibitor failure patients. Compared with existing treatment regimens, CDK2 inhibitors are more targeted in their mechanism of action, but their efficacy may be weaker than that of ADC drugs. Compared with CDK4/6 inhibitors, its applicable population is narrower but more targeted.

#### 3.3.3 Dual-pathway inhibition

In patients with HR+/HER2- ABC who have failed CDK4/6i treatment, the dual-pathway inhibition strategy provides a new approach to overcoming complex drug resistance mechanisms by synergically targeting the PI3K/AKT/mTOR and ER signaling pathways. The core of this strategy lies in blocking the compensatory signal activation of tumor cells after CDK4/6i, tumors often escape through the upregulation of the PI3K/AKT/mTOR pathway or ESR1 mutations, and dual-pathway inhibition can effectively suppress these bypass signals. For instance, TRINITI-1 (NCT02732119) was a Phase I/II study evaluating the combination of exemestan, ribociclib and everolimus. The results showed a mPFS of 7.4 months, a clinical benefit rate of 41.8%, and more significant benefits in the ESR1 mutant subgroup (median PFS of 8.1 months). However, the objective response rate was relatively low (13.9%). In terms of safety, the incidence of grade 3 or higher neutropenia was 46%, grade 3 stomatitis was 18%, and 42% of patients required a reduced dose of ribociclib. This suggests that although this regimen offers a non-chemotherapy option for patients resistant to CDK4/6i, toxicity management remains a significant challenge. This type of “multi-target coverage” (38) protocol can delay the occurrence of drug resistance, especially suitable for patients with multi-drug resistance or rapid progression. Therefore, the 2024 NCCN guidelines list some dual-target protocols as Class II recommendations for specific molecular subgroups (such as PIK3CA mutations). However, the accumulation of toxicity has led to the need for dose reduction in 30-40% of patients. Therefore, in the future, it is still necessary to optimize the administration strategy and precisely screen the beneficiary population.

### 3.4 The research significance of second-line treatment for failed CDK4/6i therapy

Although CDK4/6i has become a major advance in the treatment of HR+ breast cancer, the emergence of drug resistance has led to treatment failure in some patients, making the exploration of subsequent therapeutic options an urgent priority. Research into post-resistance treatment holds several key implications: First, it addresses unmet clinical needs-current standard treatment remains unclear, and the efficacy and safety of alternative endocrine therapies, targeted combinations (such as mTOR/PI3Ki), chemotherapy, or novel agents (e.g., AKTi/SERD) require systematic evaluation. Second, it helps elucidate resistance mechanisms (such as RB1 loss, cyclin E-CDK2 activation, etc.), thereby facilitating the development of precise combination strategies. Third, it enables the optimization of treatment sequencing based on resistance heterogeneity to achieve individualized stratification. Fourth, it accelerates the research and development of new drugs (e.g., CDK2/4/6 (16) dual-target inhibitors, PROTAC (17, 38) degraders). In summary, such research may help to prolong patient survival and improve quality of life, while also deepening the understanding of resistance mechanisms and promoting the development of precision therapy.

### 3.5 Therapeutic sequencing considerations after CDK4/6i failure

The optimal sequencing strategy after CDK4/6i failure remains incompletely defined, as no head-to-head trials have directly compared different post-CDK4/6i strategies. Treatment selection should therefore be individualized based on three major factors: (1) the mechanism of resistance (endocrine-sensitive vs. endocrine-refractory disease), (2) actionable genomic alterations (e.g., PIK3CA, ESR1, AKT1, PTEN), and (3) disease burden and tempo of progression.

In patients with ESR1 mutations and retained endocrine sensitivity, oral SERDs represent a rational option; however, most available data are derived from molecularly selected populations, and their benefit outside this context remains uncertain. For tumors harboring PIK3CA mutations, PI3K inhibitors are supported by randomized evidence, although toxicity—particularly hyperglycemia and gastrointestinal events—may limit real-world applicability. AKT inhibitors have shown activity in biomarker-enriched subgroups, but their optimal placement in the treatment sequence remains to be clarified. Beyond molecular selection, the clinical pattern of disease progression should also guide therapeutic choice. Patients with indolent, non-visceral progression may reasonably continue endocrine-based strategies, whereas those with rapid progression or visceral crisis may require therapies associated with higher objective response rates, such as antibody–ADCs or chemotherapy. Prior treatment tolerability further refines decision-making. Significant metabolic toxicity with prior PI3K inhibition, cumulative hematologic toxicity from CDK4/6 inhibitors, or patient comorbidities may influence the feasibility of subsequent targeted approaches in routine practice. Importantly, potential cross-resistance within the PI3K/AKT/mTOR axis should be considered, as sequential targeting of the same pathway may yield diminishing returns. Given the heterogeneity of resistance mechanisms and the absence of universally accepted sequencing algorithms, alternating therapeutic mechanisms and incorporating dynamic biomarker assessment represent a pragmatic, evidence-informed strategy in current clinical practice.

## 4 Summary and future clinical challenges

The introduction of CDK4/6i has substantially improved outcomes in HR+/HER2− advanced breast cancer; however, acquired resistance remains inevitable for most patients. As no universally accepted post-CDK4/6i sequencing algorithm exists, treatment decisions must integrate resistance mechanisms, biomarker status, and the clinical pattern of disease progression.

Emerging therapeutic strategies—including next-generation endocrine therapies, PI3K/AKT/mTOR-targeted agents, and antibody–drug conjugates—offer biologically rational options in selected patient populations, yet optimal sequencing and cross-resistance patterns remain incompletely defined. Prospective biomarker-driven trials and real-world evidence are therefore essential to refine treatment strategies and validate predictive markers.

Future advances will likely depend on improved molecular stratification, including dynamic monitoring of alterations such as ESR1 and PIK3CA using ctDNA, and better characterization of tumor evolution through multi-omics approaches. Innovative clinical trial designs, such as adaptive platform studies, may further accelerate therapeutic optimization. Ultimately, the management of HR+/HER2− advanced breast cancer after CDK4/6i progression requires a pragmatic, biology-informed, and patient-centered approach aimed at balancing efficacy, tolerability, and quality of life.
